# Mesozeaxanthin protects the liver and reduces cardio-metabolic risk factors in an insulin resistant rodent model

**DOI:** 10.1080/16546628.2017.1353360

**Published:** 2017-07-18

**Authors:** Kazim Sahin, Cemal Orhan, Fatih Akdemir, Mehmet Tuzcu, Nurhan Sahin, Ismet Yilmaz, Shakir Ali, Jayant Deshpande, Vijaya Juturu

**Affiliations:** ^a^ Department of Animal Nutrition, Faculty of Veterinary Science, Firat University, Elazig, Turkey; ^b^ Department of Nutrition, Faculty of Fisheries, Inonu University, Malatya, Turkey; ^c^ Division of Biology, Faculty of Science, Firat University, Elazig, Turkey; ^d^ Department of Pharmacology, Faculty of Pharmacy, Inonu University, Malatya, Turkey; ^e^ Department of Biochemistry, Faculty of Science, Jamia Hamdard, New Delhi, India; ^f^ Research and Development, OmniActive Health Technologies Inc., Morristown, NJ, USA

**Keywords:** High-fat diet, insulin resistance, liver, mesozeaxanthin, rats

## Abstract

**Background:** Mesozeaxanthin (MZ) is a macular carotenoid which has been reported to have a number of pharmacological properties, including the antioxidant, and anticarcinogenic property, and has been stated to decrease the hepatocyte lipid content.

**Objective:** In this study, we investigated the effect of MZ on cardio-metabolic health risk (CMHR) and its probable mechanisms of action in rats fed a high-fat diet (HFD).

**Design:** Rats were randomly divided into four groups consisting of (i) Control, (ii) MZ, (iii) HFD, and (iv) HFD+MZ.

**Results:** MZ treatment increased the antioxidant enzyme activities and helped improve the liver function. The treatment alleviated CMHR and decreased the level of nuclear factor kappa B (NF-κB p65) and tumor necrosis factor-alpha (TNF-α). The levels of hepatic peroxisome proliferator-activated receptor gamma (PPAR-γ), phosphorylated insulin receptor substrate 1 (p-IRS-1), β,β-carotene 9’,10’-oxygenase 2 (BCO2) and nuclear factor erythroid 2-related factor 2 (Nrf2), which decrease in HFD rats, were found to be significantly higher in MZ supplemented animals.

**Conclusion:** MZ has antioxidant and anti-inflammatory properties and can is reported in this study toprotect against fatty liver and cardio-metabolic syndrome, possibly through regulation of PPAR-γ, IRS-1, Nrf2 and NF-κB proteins, in an insulin-resistant rodent model.

The cardio-metabolic syndrome (CMS) has been reported to be related to the risk of developing type 2 diabetes mellitus (T2DM), cardiovascular disease (CVD) and stroke [1–4]. The syndrome, characterized by the clustering of at least three of five of the following medical conditions – (i) central or abnormal obesity; (ii) high blood pressure (BP); (iii) raised fasting plasma glucose; (iv) high plasma triglycerides (TG); and (v) low level of high-density lipoprotein cholesterol (HDL-C) – has been reported to be caused by excess adiposity as a usual key factor [3,4]. Fatty liver in obese individuals is one of the most critical cardio-metabolic health risk (CMHR) in insulin resistance, thus leading to CMS [5,6]. In the liver, existence of some fat is normal, but an increase in fat content to more than 5–10% of the liver weight has been associated with the fatty liver, which can be alcoholic or non-alcoholic and may lead to serious complications related to lipid imbalance and increased free fatty acids in the blood [7,8].

Mesozeaxanthin [MZ; (3R,30S)-dihydroxy-b, b-carotene-3,30-diol] is a non-provitamin A carotenoid in green and yellow vegetables which has been found to be effective in aging macula to maintain its structural density [9,10]. The pigment resides directly over the centre of macula where there is the strongest need for protection against actinic blue light. The pigment is synthesized in the retina from ingested lutein. If taken as a supplement, MZ is reported to be absorbed into the blood stream and effectively increase the macular pigment concentration. The macular pigment has been reported to have various protective effects which include antioxidant, chemo-protective, anti-mutagenic and anti-carcinogenic effects, owing to its singlet oxygen quenching ability and its inhibitory effect on specific CYP450 proteins, besides its potential to induce phase II enzymes [9–12]. *In vitro*, MZ has been demonstrated to reduce hepatocyte lipid content [10,13,14], which led us to speculate on the potential of MZ in reducing fatty liver, a CMHR. We tested this hypothesis in a rat model of insulin resistance (IR) induced by high-fat diet (HFD) in rodents. This study showed a significant correlation between MZ supplementation and decreased the risk of fatty liver and CMS and has implication in the management of fatty liver and insulin resistance by regulating PPAR-γ, IRS-1, NF-κB and Nrf2 pathways.

## Material and methods

### Animals and diets

Eight weeks old male Sprague-Dawley rats (180 ± 20 g) were used in this study. The rats were kept at 22 ± 2 °C, 55 ± 5% humidity and a 12/12 h light/dark cycle and provided rat chow (Research Diet, NJ, USA) and water *ad libitum*. All rats received humane care according to the standards defined in the ‘Guide for the Care and Use of Laboratory Animals’ prepared by the National Academy of Sciences and published by National Institute of Health. The study was permitted by the Ethics Committee of the University of Inonu, Malatya, Turkey. The composition of control and high-fat diet [15] is shown in detail in Table 1. For induction of obesity, animals were fed with HFD for 12 weeks and compared with normal diet fed rats.

**Table 1.** Composition of diets (g/kg diet) fed to rats.

	Control	HFD
Casein	200.0	200.0
Starch	579.5	150.0
Sucrose	50.0	149.5
Soybean oil	70.0	–
Beef tallow	–	400.0
Cellulose	50.0	50.0
Vitamin–mineral premix*	45.0	45.0
l-cysteine	3.0	3.0
Choline bitartrate	2.5	2.5

Notes: *The vitamin–mineral premix provides the following (per kg): all-*trans*-retinyl acetate, 1.8 mg; cholecalciferol, 0.025 mg; all-*rac*-a-tocopherol acetate, 12,5 mg; menadione (menadione sodium bisulfate), 1.1 mg; riboflavin, 4.4 mg; thiamine (thiamine mononitrate), 1.1 mg; vitamin B-6, 2.2 mg; niacin, 35 mg; Ca-pantothenate, 10 mg; vitamin B-12, 0.02 mg; folic acid, 0.55 mg; *d*-biotin, 0.1 mg. manganese (from manganese oxide), 40 mg; iron (from iron sulfate), 12.5 mg; zinc (from zinc oxide), 25 mg; copper (from copper sulfate), 3.5 mg; iodine (from potassium iodide), 0.3 mg; selenium (from sodium selenite), 0.15 mg; choline chloride, 175 mg.

### Experimental procedures

**Figure 1.**
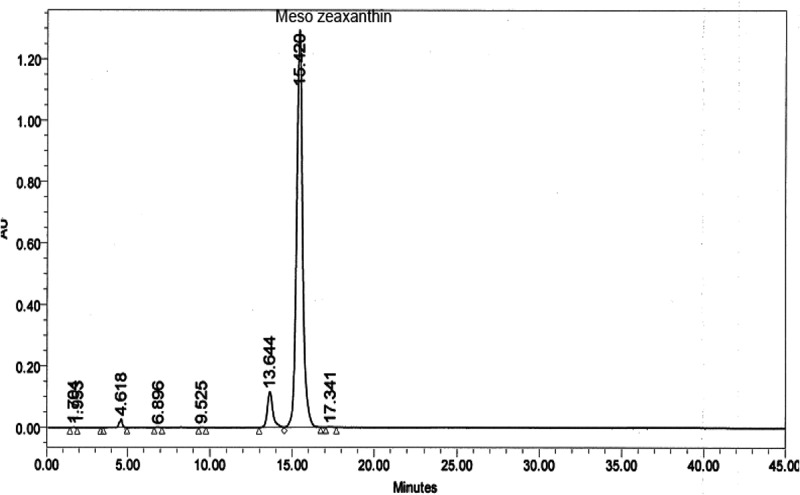
Chromatography (HPLC) of mesozeaxanthin.

Twenty-eight animals were acclimatized for two weeks and randomly divided into four groups (n = 7), consisting of (i) control group (untreated rats, fed with normal chow, 12% of calories as fat, for 12 weeks), (ii) MZ supplemented group (fed normal chow and treated with MZ, 100 mg/kg BW, for 12 weeks), (iii) HFD group (rats fed a high-fat diet, 42% of calories as fat, for 12 weeks), and (iv) HFD+MZ group (treated with high-fat diet and MZ for 12 weeks). MZ (5%) was dissolved and delivered in sunflower oil orally by gavage. The selection of MZ dose (100 mg/kg BW) was based on previously published studies, where the compound has been shown to exhibit a significant antioxidant effect in rodents [11,16]. The control rats in this study received similar amounts of sunflower oil by gavage. MZ was supplied by Omni Active Health Technologies Pvt. Ltd. (Mumbai, India). The composition consisted of 92% (trans,3R,3′S,meso)-zeaxanthin out of the total xanthophyll content. The amount of (trans, R,R)-zeaxanthin was found to be 4% and the amount of (trans, R,R)-lutein was found to be 4% based on peak areas. The composition of other ingredients was enantiomers, metabolites, esters, salts, derivatives either alone, or in combinations thereof, along with one or more food grade excipients. The result of chromatographic analysis of MZ is shown in Figure 1. At the end of the study, the blood was collected after an overnight fast and all animals were sacrificed by cervical dislocation. The visceral fat and liver samples were removed and weighed after sacrificing the animals.

### Biochemical estimations

Serum was prepared by centrifuging the blood at 3000 × *g* for 10 min and used for the analyses of glucose, insulin, and malondialdehyde (MDA). Serum parameters were measured using an automatic analyser (Samsung LABGEO^PT10^, Samsung Electronics Co, Suwon, Korea). Repeatability and device/method exactness of LABGEO^PT10^ was checked according to the IVR-PT06 guideline. Serum insulin, leptin, and adiponectin were measured with the Rat Insulin, Leptin and Adiponectin Kits (Linco Research Inc., St Charles, MO, USA) by ELISA (Elx-800, Bio-Tek Instruments Inc., Vermont, USA) respectively. The sensitivity of the assays was 0.22, 0.36 and 0.15 ng/ml for insulin, leptin, and adiponectin respectively. The interassay and intra-assay coefficients of variation were 4.1 and 6.7% for insulin, 2.9 and 5.8% for leptin, and 2.5 and 6.4% for adiponectin respectively. Insulin resistance was assessed using the homeostasis model assessment for insulin resistance (HOMA-IR), which was calculated using the following formula: glucose (mg/dl) × insulin (μIU/ml)/22.5.

The macular carotenoids in the hepatic tissue (MZ, Z, and L) were determined by high-performance liquid chromatography (HPLC) (Shimadzu, Tokyo, Japan) using Shimadzu UV-vis (UV-visible Spectrophotometer) SPD-10AVP detector and C18−ODS-3, 5 µm, 4.6 × 250 mm column. Briefly, 30 mg of the liver tissue was added to a mixture of 100 µL 12% pyrogallol in ethanol, 200 µL echinenone (43 µg/dL) as internal standard, 200 µL 30% KOH, 60 µL butylated hydroxytoluene (BHT) (10 mg/mL) and 1 mL ethanol. The samples were then saponified at 37°C for 2 h. The mixture was extracted twice with 3 mL ether: hexane (2:1, by vol.). The extract was evaporated to dryness under nitrogen. The residue was dissolved in 150 µL ethanol, and a 50 µL sample was used for HPLC analysis [17]. All extracts were protected on the ice and from light. In the HPLC analysis, the column was passed through with methanol:acetonitrile:dichloromethane:water (7:7:20:0.16) containing 20 mmol/L ammonium acetate at a flow rate of 1 ml per min and detection at 450 nm.

The hepatic MDA content was determined by HPLC according to the method described by Karatepe [18] using a Shimadzu UV–vis SPD-10 AVP detector, a CTO-10 AS VP column and a mobile phase consisting of 30 mM KH_2_PO_4_ and methanol (82.5: 17.5, v/v, pH 3.6) at a flow rate of 1.2 ml/min. Column effluents were monitored at 250 nm. The volume of the sample injected into HPLC was 20 μl. Liver homogenate (10%, w/v) was prepared in 10 mM phosphate buffer (pH 7.4), centrifuged at 13,000 × *g* for 10 min at 4 °C. The resulting supernatant was collected and stored at −80°C for the estimation of MDA.

Total antioxidant capacity (TAC) was determined by following the reduction of dark blue-green 2,2′-azino-bis 3-ethylbenzothiazoline-6-sulfonate (ABTS) dye into a colourless form by antioxidants [19]. The dye ABTS was oxidized by incubating with potassium persulfate. Briefly, 10 mg ATBS was dissolved in 10 mL of an aqueous solution of 2.5 mmol/L potassium persulfate and allowed to stand in the dark at room temperature for one to four hours before use. For use, ABTS oxidized stock solution was diluted with deionized water to an absorbance of 0.70 at 734 nm. After the addition of 1 mL of diluted ABTS oxidized to 10 µL of serum, the absorbance reading was taken 10 minutes after initial mixing. The assay has been reported for its excellent precision (within- and between-laboratory precision values are reported to be lower than 3%). The results were expressed in mmol Trolox equivalents/L.

Total superoxide dismutase (SOD) activity (cytosolic and mitochondrial) in the homogenized tissue (in 20 mM HEPES (N-2 hydroxy-ethyl piperazine-N’-2-ethanesulfonic acid) buffer, 1 ‎mM ethylene glycol tetraacetic acid, 210 mM mannitol, 70 mM sucrose, pH 7.2 per g of tissue) was measured using a commercial kit (Cayman Chemical, Ann Arbor, MI, USA) according to the manufacturer’s instructions. The supernatant was ‎collected after ‎centrifugation at ‎12.000 *g* for 20 min at 4°C‎. The supernatant was desalted by passage through a Sephadex G-25 column. The SOD samples were also treated with a mixture of ethanol-chloroform (2:1, v/v) and distilled water to eliminate hemoglobin and red blood cells and then the absorption was read on a plate reader (Biotek Instruments, Inc., Vermont, USA) at 450 nm. Results were expressed as units per mg of protein (U/mg of protein). Catalase (CAT) activity was also determined in the homogenized tissue (in cold buffer containing 50 mM ‎potassium ‎phosphate, 1 mM EDTA, pH 7, per g of tissue) using a commercial kit (Cayman Chemical, Ann Arbor, MI, USA) according to the manufacturer’s instructions. The supernatant was collected after ‎centrifugation at ‎12,000 *g* for 20 ‎min at 4 °C. A formaldehyde solution was used as a standard. The absorbance of standard and samples was taken at 540 nm using a plate reader (Biotek Instruments, Inc., Vermont, USA). Catalase activity was expressed in nmol/min/mg of protein. Glutathione peroxidase (GSHPx) was assayed according to the manufacturer´s instructions (Cayman Chemical, Ann Arbor, MI, USA). Liver tissue was homogenized with the polytron homogenizer in cold buffer ‎(50 mM ‎Tris-‎HCL, PH 7.5, 5 mM ‎EDTA, and 1 mM dithiothreitol)‎ per g tissue and then centrifuge at 10,000 *g* for ‎‎15 ‎min at 4°C‎. This method is based on the oxidation of NADPH to NADP^+^, which is accompanied by a decrease in absorbance at 340 nm. GSHPx activity was measured by initiating the reaction with 2.4 mM cumene hydroperoxide. One unit is defined as the amount of enzyme that oxidizes 1 μmol of NADPH per min at 25°C. The absorbance was read every minute at 340 nm using a plate reader (Biotek Instruments, Inc., Vermont, USA) to obtain at least five time points. The GSHPx activity was calculated in nmol/min/mg of protein.

### Western blot analyses

For Western blot, protein extraction was done by standardizing the liver in 1 ml ice-cold hypotonic buffer (buffer A), comprising 10 mM HEPES (2-(4-(2-hydroxyethyl)-1-piperazinyl) ethane sulfonic acid), pH 7.8, 10 mM KCl, 2 mM MgCl_2_, 1 mM dithiothreitol (DTT), 0.1 mM EDTA, and 0.1 mM phenylmethylsulfonyl fluoride (PMSF). The homogenate was mixed with 80 μl of 10% Nonidet P-40 (NP-40) solution and then centrifuged at 14,000 × *g* for 2 min. The precipitates were washed once with 500 μl of Buffer-A plus 40 μl of 10% NP-40, centrifuged and re-suspended in 200 μl of a buffer containing 50 mM HEPES, pH 7.8, 50 mM KCl, 300 mM NaCl, 0.1 mM EDTA, 1 mM DTT, 0.1 mM PMSF, and 20% glycerol), and re-centrifuged at 14,800 × *g* for 5 min. The supernatant was collected and used for the determination of NF-κB p65, TNF-α, BCO2, IRS-1, PPAR-γ, Nrf-2 and HO-1, according to the method defined previously [20]. Briefly, 50 μg of proteins were electrophoresed and then transported onto a nitrocellulose membrane (Schleicher and Schuell Inc., Keene, NH, USA). Antibodies against NF-κB p65, TNF-α, BCO2, IRS-1, PPAR-γ, Nrf-2 and HO-1 (Abcam, Cambridge, UK) were diluted (1:1000) in the same buffer containing 0.05% Tween-20. The phosphorylated forms of the antibodies [anti-NF-kB p65 antibody (ab16502); anti-PPAR gamma antibody (ab209350) and anti-Nrf2 antibody (ab31163)] were used in this study. Protein loading was controlled using monoclonal mouse antibody against β-actin (A5316; Sigma). Bands were examined densitometrically using ImageJ, an image analysis system (National Institute of Health, Bethesda, USA).

### Statistical analysis

All values were expressed as mean ± SE. The difference between groups was evaluated by the GLM procedure of SAS (SAS Institute: SAS User’s Guide: Statistics, 2002). The treatments were compared using ANOVA and Student’s unpaired *t*-test, and P < 0.05 was considered statistically significant.

## Results

### Effect of MZ on metabolic health markers

HFD caused a significant increase in visceral fat and liver weight (Table 2); both of which were reduced in MZ supplemented rats. The percentage increase in body weight of 146.7% in HFD rats was reduced to 139% in MZ supplemented group. There was also a substantial increase registered in the liver weight to body weight ratio (data not shown) of HFD-fed rats compared to controls and MZ-treated groups. MZ supplementation also caused a decrease in liver weight.

**Table 2.** Effect of MZ supplementation on the body weight, visceral fat and liver weight in rats fed HFD for 12 weeks.

	Groups			
Item	Control	MZ	HFD	HFD + MZ
Initial BW (g)	233.86 ± 5.42	234.43 ± 3.62	232.29 ± 3.93	234.43 ± 5.74
Final BW (g)	286.29 ± 5.65^b^ (122%)	291.71 ± 6.48^b^ (124.4%)	340.86 ± 5.00^a^ (146.7%)	326.29 ± 8.25^a^ (139%)
Feed intake (g/d)	23.54 ± 0.49^a^	23.31 ± 0.40^a^	19.16 ± 0.44^b^	19.28 ± 0.78^b^
Visceral fat (g)	7.79 ± 0.25^c^	7.69 ± 0.26^c^	29.54 ± 2.20^a^	15.33 ± 0.30^b^
Liver (g)	12.29 ± 0.21^c^ (4.29%)	11.84 ± 0.28^c^ (4.05%)	20.56 ± 0.60^a^ (6.03%)	17.19 ± 0.79^b^ (5.26%)

Notes: MZ, mesozeaxanthin; HFD, high-fat diet; data are expressed as mean±SEM of 7 rats from each group. Superscripts a–c indicate that means in the same row without a common superscript differ signiﬁcantly (P < 0.05).

### Effect of MZ on biochemical markers of carbohydrate and lipid metabolism and the liver and kidney functions tests in HFD rats with or without MZ supplementation

There was a significant rise in glucose, insulin, HOMA-IR, free fatty acid and leptin in HFD-fed rats (Table 3). MZ supplementation caused a considerable decrease in increased levels of these substances. The study reports an almost 50% decrease in adiponectin, and an increase in total cholesterol, HDL-C and LDL-C, as well as TG in HFD rats. The changes in these biochemical indicators of lipid metabolism were brought down to normal by MZ supplementation. HFD did not cause a significant increase in aspartate transaminase (AST), alanine transaminase (ALT), alkaline phosphatase (ALP), gamma-glutamyltransferase (GGT), urea (BUN) and creatinine (CRE) in the duration of the treatment, and the levels remained more or less unaffected in MZ-supplemented rats. Albumin and total protein also remained unaffected in HFD alone or MZ-supplemented animals fed with HFD.

**Table 3.** Effects of MZ on biochemical parameter levels in rats fed HFD for 12 weeks.

Item	Groups			
	Control	MZ	HFD	HFD + MZ
Glucose (mmol/L)	4.38 ± 0.23^c^	4.45 ± 0.24^c^	10.83 ± 0.29^a^	9.19 ± 0.11^b^
Insulin (pmol/L)	306.34 ± 10.33^c^	285.69 ± 12.05^c^	1335.50 ± 30.98^a^	829.52 ± 29.26^b^
HOMA-IR	8.60 ± 0.35^c^	8.14 ± 0.26^c^	92.66 ± 4.52^a^	48.83 ± 2.84^b^
FFA (mM)	1.23 ± 0.09^c^	1.02 ± 0.06^c^	3.81 ± 0.23^a^	2.03 ± 0.06^b^
Leptin (ng/mL)	30.71 ± 2.88^c^	29.57 ± 3.09^c^	107.71 ± 3.93^a^	75.43 ± 3.95^b^
Adiponectin (mg/mL)	10.37 ± 0.28^a^	11.02 ± 0.39^a^	5.97 ± 0.07^c^	8.63 ± 0.20^b^
T-C (mmol/L)	1.32 ± 0.02^b^	1.27 ± 0.02^b^	2.15 ± 0.09^a^	1.41 ± 0.03^b^
HDL-C (mmol/L)	0.44 ± 0.01^bc^	0.38 ± 0.02^c^	0.54 ± 0.03^a^	0.50 ± 0.01^ab^
LDL-C (mmol/L)	0.69 ± 0.02^bc^	0.63 ± 0.09^c^	1.20 ± 0.08^a^	0.86 ± 0.01^b^
TG (mmol/L)	0.31 ± 0.01^c^	0.27 ± 0.03^c^	0.57 ± 0.03^a^	0.41 ± 0.02^b^
AST (U/L)	138.43 ± 7.80	135.00 ± 6.66	143.29 ± 6.61	134.14 ± 3.00
ALT (U/L)	72.71 ± 5.55	75.71 ± 4.99	77.29 ± 2.08	73.86 ± 3.83
ALP (U/L)	2012.00 ± 69.25	1989.86 ± 83.34	1983.86 ± 105.63	2060.14 ± 80.91
Albumin (g/L)	30.35 ± 0.50	29.63 ± 1.07	30.91 ± 0.83	29.96 ± 0.78
Total protein (g/L)	59.74 ± 1.25	60.49 ± 0.81	60.55 ± 1.15	61.00 ± 0.98
Bilirubin (µmol/L)	3.08 ± 0.17	3.93 ± 0.34	3.25 ± 0.19	3.76 ± 0.21
GGT (U/L)	9.98 ± 0.01	9.97 ± 0.03	9.98 ± 0.04	9.97 ± 0.02
BUN (mmol/L)	3.55 ± 0.19	3.47 ± 0.44	3.72 ± 0.35	3.23 ± 0.20
CRE (µmol/L)	7.07 ± 0.18	6.63 ± 0.11	7.43 ± 0.23	6.36 ± 0.27

Notes: MZ, mesozeaxanthin; HFD, high-fat diet; FFA, free fatty acids; T-C, total cholesterol; HDL-C, high-density lipoprotein cholesterol; LDL-C, low-density lipoprotein cholesterol; TG, triglycerides; AST, aspartate aminotransferase; ALT, alanine transferase; ALP, alkaline phosphatase; GGT, gamma-glutamyltransferase; BUN, blood urea nitrogen; CRE, creatinine. Data are expressed as mean±SEM of 7 rats from each group. Superscripts a–c indicate that means in the same row without a common superscript differ signiﬁcantly (*P* < 0.05).

### Effect of MZ on the hepatic antioxidant enzymes and carotenoid levels

As shown in Table 4, MZ supplementation caused a significant reduction in both serum and hepatic lipid peroxidation. The serum TAC, liver SOD,CAT and GSHPx decreased in HFD-fed rats, but increased in MZ-supplemented rats, although the increase was not to the level of control values. The activity of CAT was also decreased in HFD rats and increased in HFD rats supplemented with MZ. Hepatic MZ, Z and L were also measured and are shown in Table 2. Macular carotenoids could be detected only in rats administered with MZ. The hepatic concentration of MZ, Z, and L was higher in the MZ-supplemented group than in the group fed HFD+MZ diet (*P* < 0.001) (Table 4).

**Table 4.** Effects of MZ on the antioxidant status rats fed HFD for 12 weeks.

Item	Groups			
	Control	MZ	HFD	HFD + MZ
Serum MDA (nmol/mL)	0.87 ± 0.03^c^	0.73 ± 0.03^c^	1.88 ± 0.07^a^	1.56 ± 0.06^b^
Liver MDA (nmol/mg protein)	1.82 ± 0.06^c^	1.63 ± 0.09^c^	3.82 ± 0.06^a^	2.84 ± 0.12^b^
Serum TAC (nmol Trolox Equiv. per mg protein)	1.18 ± 0.09^a^	1.28 ± 0.11^a^	0.46 ± 0.05^b^	0.74 ± 0.04^b^
Liver SOD (U/mg protein)	182.86 ± 4.61^a^	196.57 ± 4.42^a^	106.29 ± 2.50^c^	134.43 ± 2.52^b^
Liver CAT (U/mg protein)	299.71 ± 8.45^ab^	313.57 ± 9.80^a^	253.43 ± 9.42^c^	276.43 ± 9.66^bc^
Liver GSHPx (U/mg protein)	41.29 ± 0.94^b^	53.14 ± 2.40^a^	16.86 ± 1.16^d^	26.71 ± 3.54^c^
Zeaxanthin *+ meso*-zeaxanthin (mg/g)	-	6.36 ± 0.54^a^	-	4.52 ± 0.64 ^b^
Lutein (mg/g)	-	8.23 ± 1.45^a^	-	5.88 ± 1.04 ^b^

Notes: MZ, mesozeaxanthin; HFD, high-fat diet; MDA, malondialdehyde; TAC, total antioxidant capacity; SOD, superoxide dismutase; CAT, catalase; GSHPx, glutathione peroxidase. Data are expressed as mean±SE of 7 rats from each group. Superscripts a–c indicate that means in the same row without a common superscript differ signiﬁcantly (P < 0.05).

### Effect of MZ on the level of inflammatory mediators and the protein involved in the detoxification process

**Figure 2.**
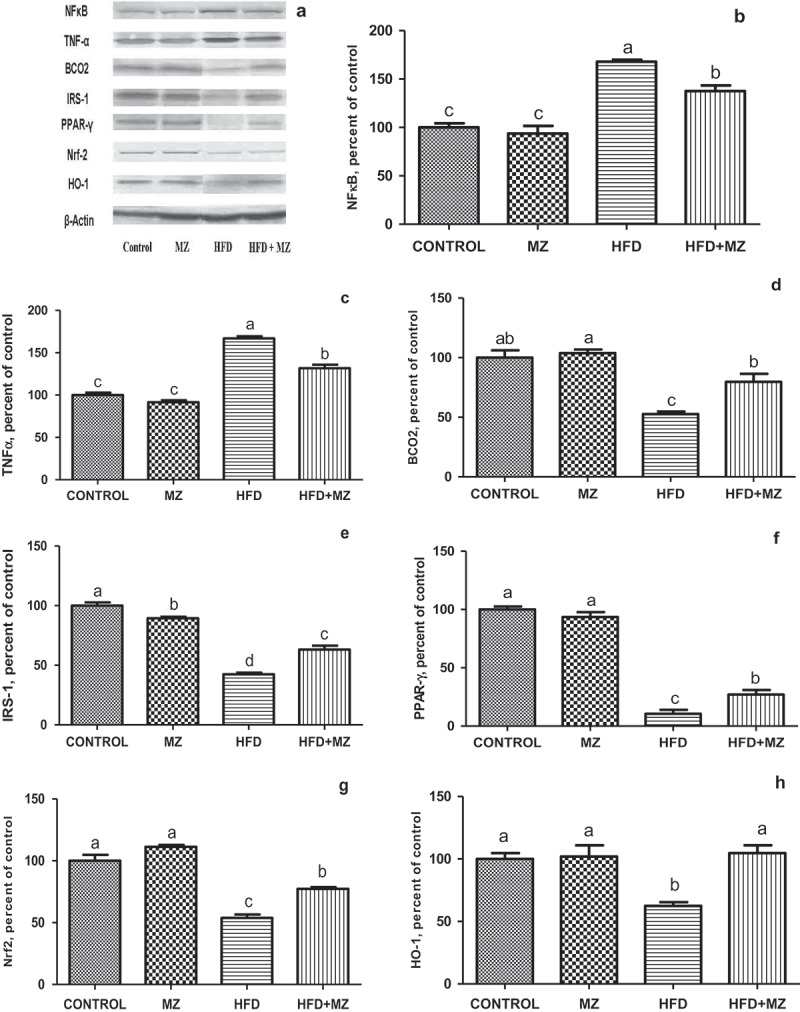
Hepatic NF-κB, TNF-α, BCO2, IRS-1,PPAR-γ Nrf-2 and HO-1 expression levels in mesozeaxanthin (MZ) supplemented high-fat diet (HFD) fed rats and control groups. Notes: The Western blot strips of the proteins measured in this study are shown in Panel A. Panels B–F show the expression level of NF-κB, TNF-α, BCO2, IRS-1, PPAR-γ, Nrf-2 and HO-1 in various groups. The intensity of the bands shown in Panel A was quantiﬁed by densitometric analysis. Data are expressed as a ratio of normal control value (set to 100%). Each bar represents the mean and standard error of mean. Blots were repeated at least three times (n = 3) and only a representative blot is shown in Panel A. β-Actin was included to ensure equal protein loading.

MZ reduced the elevated level of hepatic NF-κB p65 and TNF-α in HFD-fed rats (Figure 2 Panels A–C). In rats treated with MZ alone, the expression level of these proteins was the same as that of the control group. When compared with the untreated HFD group, MZ caused a significant increase in liver BCO2, IRS-1, PPAR-γ (Figure 2 Panels D–F), Nrf-2 and HO-1 (Figure 2 Panels G and H) in HFD rats. MZ supplementation resulted in a near normalization of the expression of HO-1 in HFD-fed rats (Figure 2, Panel H).

## Discussion

Prevalence of obesity predisposes to metabolic syndrome, which may lead to fatty liver and chronic conditions including T2DM, CVD and stroke. In morbidly obese people, about 90% show histological defects of the liver and one-third of these patients have fatty alteration involving more than 50% hepatocytes [6,21]. HFD-induced obesity is reported to impair carbohydrate and lipid metabolism [6]. In this study, we observed a significant increase in visceral fat and liver weight in rats fed HFD. The changes were significantly brought down in MZ-supplemented animals, suggesting an overall beneficial effect of MZ on obesity-related parameters, thus indicating its possible role in preventing the metabolic syndrome. MZ supplementation also caused a significant decrease in glucose, insulin, HOMA-IR, free fatty acid and leptin in HFD fed rats when compared with the non-supplemented HFD rats. It also increased adiponectin and reduced cholesterol, LDL-C and TG. There was no significant change in HDL-C when the MZ-supplemented group was compared with HFD-fed rats. Adiponectin is particularly important for its role in lipid metabolism and its increase in MZ-supplemented animals suggests a protective role of adiponectin on fatty liver and hence associated complications. In this study, MZ treatment did not produce any sign of toxicity, as indicated by the biochemical markers of hepatic injury. The finding is consistent with the literature, where MZ is not reported for toxicity [22]. The serum albumin and total protein in this study also remained unaffected. In the current study, 100 mg/kg dosage was chosen on the basis of earlier published work where MZ at this dose level showed a significant antioxidant effect in rodents [11,16]. With regard to the effect on hepatic markers, as reported by Firdous et al. [11], MZ (50 and 250 mg/kg BW) caused a near normalization of the level of hepatic markers of injury in nitroso diethylamine (NDEA)-induced hepatocellular carcinoma in male Wistar rats. The study reported a significant increase in the levels of γ-glutamyl transpeptidase, alanine aminotransaminase, aspartate aminotransferase and alkaline phosphatase in NDEA-treated animals in both serum and the liver in a dose-dependent manner, which was significantly brought down by MZ. The study also reported a decrease in oxidative stress markers (lipid peroxidation, conjugated dienes and tissue hydroperoxides) by MZ and an increase in glutathione and antioxidant enzymes (superoxide dismutase, catalase, and glutathione peroxidase) which can be attributed to the antioxidant activity of macular carotenoid in HFD fed rats. The effect on oxidative stress markers suggests the protection of the liver against reactive oxygen species generated as a result of consumption of HFD. Earlier, MZ at a dose level of 100 mg/kg BW has been reported by Orhan et al. [16] to protect the retina in HFD fed rats by counteracting the effects of oxidants in the retina and regulating growth and transcription factors.

Oxidative stress has been suggested as a causal factor in the progression of hepatic steatosis in animals [23]. The free radicals and other reactive oxygen species produced as a result of metabolic processes in the body are counteracted by a number of mechanisms, which include the antioxidant enzymes that constitute an important line of defence against these highly reactive moieties. The second line of protection against free radical damage is provided by the antioxidants. An antioxidant is a molecule stable enough to donate an electron to a free radical and neutralize its detrimental result. Vitamins E, C and carotenoids are some of the commonly used antioxidants. MZ, the macular carotenoid, has a significant antioxidant action which can be caused by the conjugated polyene structure of carotenoids. Meso-zeaxanthin, a stereoisomer of zeaxanthin, has been reported probably to have the same antioxidant effect as zeaxanthin [11]. It has also been stated that in association with a zeaxanthin binding protein, the pi-isoform of glutathione-S-transferase, MZ provides better defence against lipid membrane oxidation than zeaxanthin [24]. The present study suggests that supplementation of MZ by its antioxidant activity to HFD-induced obese rats causes reduced MDA level and improved antioxidant enzyme (SOD and CAT) activities in the liver. Additionally, we could not find a detectable amount of Z+MZ and L in the liver of rats that were not supplemented with MZ.

In this study, we stated a noteworthy reduction in serum and liver lipid peroxidation and an augmented antioxidant response in MZ supplemented animals, supporting the strong antioxidant function of MZ in the liver. MZ, because of its ability to exhibit little or no pro-oxidative behaviour, even at high carotenoid concentration and at high oxygen tension, has been suggested to be a ‘pure’ antioxidant [25]. Studies published earlier have suggested that supplementation of MZ can scavenge superoxide and hydroxyl radicals and inhibit in vitro lipid peroxidation [10,11]. It has also been reported that oral administration of MZ inhibited superoxide radicals generated in macrophages by 25.2, 50.1 and 67.2% at three different doses of low (50), moderate (100) and high (250 mg/kg BW) respectively [11]. The present study also reported an increase in the activities of antioxidant enzymes like CAT, SOD and GSHPx in MZ-supplemented rats. These enzymes showed a decrease in HFD rats but increased in MZ supplemented animals. The results suggest a profound antioxidant effect of MZ on the liver. The reduction in lipid peroxidation and the increase in carotenoids detected in rats administered with MZ support the theory that this carotenoid ought to have a role in lessening the aberrations in the liver in treated rats by its antioxidant effect [10]. There is no earlier literature related to investigating the properties of MZ on the liver carotenoid concentrations in rats fed HFD to compare with this study.

Compounds like gingerol have earlier been shown to have anti-obesity action and are reported for their beneficial influence on lipid profile, insulin, leptin, amylase and lipase in obese rats [26]. In clinical studies, levels of carotenoids, including lutein and zeaxanthin, are reported to be low in fatty liver [27]. β,β-Carotene 9ʹ,10ʹ-oxygenase 2 (BCO2), which cleaves non-pro-vitamin carotenoids (lutein and zeaxanthin) in mitochondria of the liver cells and is recognized as a gatekeeper of mitochondrial function, has been reported to make hepatic cells more susceptible to oxidative stress in mice [28,29]. The association between carotenoid metabolism and mitochondrial biogenesis/mitophagy during the progress of obesity-associated hepatic steatosis remains uncertain [30]. In this study, we found a decrease in BCO2 in HFD rats. BCO2 increased in the MZ-supplemented animals. In a recent study [10], MZ has also been found to protect the liver and decrease cardio-metabolic health risk as insulin resistance in HFD fed rats. Differences in insulin level have recently been reported to be a risk factor of cardio-metabolic health [31], thus implicating MZ not only in diabetes but also in cardio-metabolic health.

Additional energy consumption and decreased energy expenditure has been reported to stimulate metabolic dysfunction, oxidative stress and inflammatory pathologic factors such as IL-6 and TNF-α [32]. Oxidative stress and inflammatory responses are crucial in the development of type 2 diabetes, CVD and stroke, as well as in liver injury. As discussed above, CMHR decreased in the MZ supplemented group. MZ significantly alleviated the cardio-metabolic health markers and also affected inflammatory markers in HFD rats. As shown in Figure 2, MZ by virtue of its anti-inflammatory effect [10] caused a significant reduction in expression of TNF-α and NF-κB p65 in HFD-fed rats; in MZ alone treated animals, the expression level of these proteins was the same as that of the untreated control. On the other hand, MZ induced a significant increase in IRS-1, PPAR-γ (Figure 2, E and F), Nrf-2 and HO-1 (Figure 2, G and H), in HFD rats, when compared with the untreated HFD group. As reported earlier, increased adipose tissue shows a significant role in the improvement of low-grade inflammation, which is considered by cytokine production and stimulation of inflammatory cytokine signalling pathways [33]. Pro-inflammatory cytokines have been associated with dyslipidemia and atherosclerosis [34]. MZ mediated decrease in pro-inflammatory cytokines suggests a protective effect of the substance on the high adipose condition.

IRS-1, the insulin receptor substrate 1, a signalling adapter protein which shows a significant role in transmitting signals from the insulin and insulin-like growth factor-1 receptors to PI3K/Akt and Erk MAP kinase pathways, has been reported in this study to decrease in HFD rats, but to increase in the MZ supplemented group. IRS-1 shows an important role in metabolic and mitogenic (growth promoting) pathways. IRS1 mutant mice are reported to show pronounced growth impairment but had only a mild diabetic phenotype. Increase in IRS1 in HFD-fed rats by MZ suggests a protective role in diabetes. This study further reported an increase in PPAR-γ in MZ supplemented HFD rats; PPAR-γ showed a significant decrease in HFD rats not treated with MZ. PPAR-γ, which is also recognized as the glitazone receptor, is a type II nuclear receptor that controls fatty acid storage and glucose metabolism. It stimulates lipid uptake and adipogenesis by fat cells. PPARG knockout mice have been reported to fail to generate adipose tissue when fed a high-fat diet [35]. Many naturally occurring compounds, including various polyunsaturated fatty acids (PUFA), have been reported earlier to directly bind with and activate PPAR-γ [36,37].

MZ supplementation caused a near normalization of the expression of HO-1 in HFD fed rats. It also increased the antioxidant enzymes activities (CAT, SOD, and GSHPx). Heme oxygenase (HO) catalyses the degradation of heme, thus producing biliverdin, iron and carbon monoxide. There is an inducible isoform of HO in response to heavy metals, cytokines and oxidative stress, and an increase in HO has been reported to be protective [38]. An increase in MZ supplemented HFD fed rats in HO in this study suggests a protective effect of MZ on oxidative stress. The result is further supported by an increase in the level of antioxidant parameters and a concomitant decrease in MDA, as reported in this study. The carbon monoxide released in the reaction catalysed by HO can influence vascular tone independently or influence the function of nitric oxide synthase, which has an important regulatory role in metabolism.

Nutritional supplementation has been found effective in diabetes, and also in maintaining the normal retinal function, mitochondrial homeostasis, and to decrease inflammatory mediators, and therefore represent an achievable and inexpensive adjunct therapy in diabetic patients [39]. As discussed above, increased oxidative stress and inflammatory mediators are implicated in the development of diabetes and diabetic complications, which can be prevented by antioxidants. In diabetes, the levels of lutein and zeaxanthin have been reported to decrease in the serum [39,40].

## Conclusions

Taken together, the observations in his study led us to conclude that MZ has a potential as an adjunct therapy to prevent fatty liver and improve CHR. MZ supplementation could exert their health-benefiting roles in regulating fatty liver by modulation of BCO2, PPAR-γ, IRS-1 NF-κB and Nrf2 proteins.
